# A potential signature of eight long non-coding RNAs predicts survival in patients with non-small cell lung cancer

**DOI:** 10.1186/s12967-015-0556-3

**Published:** 2015-07-17

**Authors:** Meng Zhou, Maoni Guo, Dongfeng He, Xiaojun Wang, Yinqiu Cui, Haixiu Yang, Dapeng Hao, Jie Sun

**Affiliations:** College of Bioinformatics Science and Technology, Harbin Medical University, Harbin, 150081 People’s Republic of China; Department of Interventional Radiology, The Affiliated Tumor Hospital of Harbin Medical University, Harbin, Heilongjiang 150040 People’s Republic of China; School of Life Sciences, Jilin University, Changchun, 130012 People’s Republic of China

**Keywords:** Long non-coding RNA, Non-small cell lung cancer, Overall survival, Signature

## Abstract

**Background:**

Accumulated evidence suggests that dysregulated expression of long non-coding RNAs (lncRNAs) may play a critical role in tumorigenesis and prognosis of cancer, indicating the potential utility of lncRNAs as cancer prognostic or diagnostic markers. However, the power of lncRNA signatures in predicting the survival of patients with non-small cell lung cancer (NSCLC) has not yet been investigated.

**Methods:**

We performed an array-based transcriptional analysis of lncRNAs in large patient cohorts with NSCLC by repurposing microarray probes from the gene expression omnibus database. A risk score model was constructed based on the expression data of these eight lncRNAs in the training dataset of NSCLC patients and was subsequently validated in other two independent NSCLC datasets. The biological implications of prognostic lncRNAs were also analyzed using the functional enrichment analysis.

**Results:**

An expression pattern of eight lncRNAs was found to be significantly associated with overall survival (OS) of NSCLC patients in the training dataset. With the eight-lncRNA signature, patients of the training dataset could be classified into high- and low-risk groups with significantly different OS (median survival 1.67 vs. 6.06 years, log-rank test p = 4.33E−09). The prognostic power of eight-lncRNA signature was further validated in other two non-overlapping independent NSCLC cohorts, demonstrating good reproducibility and robustness of this eight-lncRNA signature in predicting OS of NSCLC patients. Multivariate regression and stratified analysis suggested that the prognostic power of the eight-lncRNA signature was independent of clinical and pathological factors. Functional enrichment analyses revealed potential functional roles of the eight prognostic lncRNAs in tumorigenesis.

**Conclusions:**

These findings indicate that the eight-lncRNA signature may be an effective independent prognostic molecular biomarker in the prediction of NSCLC patient survival.

**Electronic supplementary material:**

The online version of this article (doi:10.1186/s12967-015-0556-3) contains supplementary material, which is available to authorized users.

## Background

Lung cancer is one of the most common human cancers and is the leading cause of cancer-related deaths among both men and women globally [1], accounting for about 27% of all cancer-related deaths. In China, lung cancer has become the primary cause of cancer-related deaths, and mortality has increased by more than four times during the past three decades [2]. The overall 5-year relative survival rate for lung cancer is low at nearly 15%, which is primarily due to principal detection at late, incurable stages and a paucity of late-stage treatments [3]. Lung cancer is generally divided into two main categories: small cell lung cancer and non-small cell lung cancer (NSCLC) accounting for approximately 80% of all lung cancers.

Long non-coding RNAs (lncRNAs), a recently discovered subclass of non-coding RNA (ncRNA), are most commonly defined as RNA transcripts longer than 200 nucleotides with little coding capacity [4, 5]. Though the functions of only a limited number of lncRNAs have been well characterized, accumulating evidence has suggested that lncRNAs participate in a wide variety of biological processes, including cell differentiation, organogenesis, chromatin modification, genomic imprinting, dosage compensation, respond to diverse stimuli and so on, by exerting their functions as four archetypes: signals, decoys, guides and scaffolds [6, 7]. lncRNAs can regulate gene expression at the post-transcriptional level via competing endogenous RNA (ceRNA) crosstalk or at the transcriptional level via *cis* or *trans* and at the epigenetic regulation level [8–10]. Recently, a number of cancer-related studies have detected many dysregulated lncRNAs associated with tumorigenesis and tumor progression in a variety of cancers [11–13]. Like protein-coding genes and miRNA, some dysregulated lncRNAs play oncogene-like roles. For instance, *HOTAIR* is an lncRNA that is overexpressed in breast tumors and significantly associated with breast cancer metastasis [14]. Overexpression of lncRNA *PCAT*-*1* is associated with poor prognosis in patients with colorectal cancer (CRC) [15]. Other well-studied lncRNAs, such as *MEGS*, *GAS5*, *LIN00312* and *LinRNA*-*p21*, have instead demonstrated tumor suppressive roles [16, 17]. For example, lncRNA *LIN00312*, which is significantly down-regulated in nasopharyngeal carcinoma (NPC), was found to be an independent contributor to NPC [18]. These findings suggest that, like protein-coding genes and miRNAs, lncRNAs could serve as diagnostic and prognostic biomarkers. Li et al. [19] measured lncRNA expression in paired tumors and adjacent normal tissues of 119 patients and identified a three-lncRNA signature that could predict the survival of patients with oesophageal squamous cell carcinoma (OSCC). Recent studies have also demonstrated emerging roles of lncRNAs in NSCLC [20]. For example, lncRNA *MALAT1* (metastasis-associated lung adenocarcinoma transcript 1) is up-regulated in NSCLC based on evidence from subtractive hybridization of cDNA libraries, and can be used as an independent prognostic marker of patient survival [21]. White and colleagues [22] found 111 differentially expressed lncRNAs between lung tumors and adjacent normal tissues, some of which have been functionally validated to be involved in cellular proliferation in vitro. Nie et al. [23] identified an lncRNA *MVIH* which is over-expressed in NSCLC tissues compared with adjacent normal tissues. Subsequent studies, integrating custom-designed gene microarray and clinical information, also discovered lncRNA signatures that were significantly associated with the survival of patients with gliolastoma multiforme [24], colorectal cancer [25] and breast cancer [26]. Other recent studies have characterized tens of lncRNAs that were identified to be associated with the presence of certain lung cancer histological subtypes [27, 28]. While the prognostic power of mRNA and miRNA signatures in various cancers is well established, the power of lncRNA signatures in predicting the survival of patients with NSCLC has not yet been investigated.

In the present study, we conducted a comprehensive study of lncRNA expression profiles across 603 NSCLC patients with clinical information by repurposing the previously published NSCLC gene expression profiles, and identified an eight-lncRNA signature associated with survival. A risk score formula was constructed based on the expression data of these eight lncRNAs in the training dataset of NSCLC patients and was further confirmed in another two independent gene expression omnibus (GEO) NSCLC patient cohorts.

## Methods

### NSCLC datasets and patient information

NSCLC microarray datasets, generated with the Affymetrix platform (HG-U133A Plus 2.0), and clinical information were obtained from the GEO database. After removing the patients without available survival information, a total of 603 patients were enrolled in this study (Table 1), including 196 patients from GSE37745 (www.ncbi.nlm.nih.gov/geo/query/acc.cgi?acc=GSE37745) [29], 226 patients from GSE31210 (www.ncbi.nlm.nih.gov/geo/query/acc.cgi?acc=GSE31210) [30] and 181 patients from GSE50081 (www.ncbi.nlm.nih.gov/geo/query/acc.cgi?acc=GSE50081) [31]. More detailed clinical information of all 603 NSCLC patients included in this study can be found in Table 1.

**Table 1:** Clinical features of all 603 NSCLC patients included in this study

Features		GSE37745 (n = 196)	GSE31210 (n = 226)	GSE50081 (n = 181)
Age (years), no (%)				
≤65		94 (48.0)	176 (77.9)	59 (32.6)
>65		102 (52.0)	50 (22.1)	122 (67.4)
Gender, no (%)				
Male		107 (55.0)	105 (46.5)	98 (54.1)
Female		89 (45.0)	121 (53.5)	83 (45.9)
Vital status (%)				
Alive		52 (26.5)	191 (84.5)	106 (58.6)
Dead		144 (73.5)	35 (15.5)	75 (41.4)
Disease stage, no (%)				
I		130 (66.0)	168 (74.3)	127 (70.2)
II		35 (18.0)	58 (25.7)	54 (29.8)
III		27 (14.0)		
IV		4 (2.0)		
Smoking status				
Never-smoker			115 (50.9)	24 (13.3)
Ever-smoker			111 (49.1)	79 (43.6)
Current				54 (29.8)
Undetermined				21 (11.6)
Histology				
Adenocarcinoma		106 (54.0)	226	128 (70.7)
Large cell carcinoma		24 (12.0)		7 (3.9)
Squamous cell carcinoma		66 (34.0)		43 (23.9)

### Microarray processing and lncRNA profile mining

All the microarray raw data (.CEL files) of three NSCLC cohorts were obtained from the GEO database and processed using the robust multichip average (RMA) algorithm for background adjustment [32, 33]. The Affymetrix GeneChip probe-level data was log-2-scale transformed and standardized by transforming the expression data into having a mean of 0 and a standard deviation (SD) of 1. The NetAffx probe set sequences for Affymetrix HG-U133 Plus 2.0 were downloaded from the Affymetrix website (http://www.affymetrix.com). LncRNA expression data from the Affymetrix-based expression profiling of NSCLC cohorts were obtained by repurposing microarray probes based on the sequences of the probe sets and the annotations of lncRNAs in GENCODE (http://www.gencodegenes.org/) (GRCh38, release 21) [34], as previous described [35]. By keeping probes that were uniquely mapped to the genomic coordinate of lncRNAs derived from GENCODE, 3,521 probes (or probe sets) and 2,313 corresponding lncRNA genes were obtained. Multiple probes (or probe sets) mapping to the same gene were integrated by using the arithmetic mean of the values of multiple probes (or probe sets) to generate a single gene expression value (on the log2 scale).

### Statistical analysis

A univariable Cox regression analysis was performed to evaluate the relationship between the continuous expression level of each lncRNA and patients’ overall survival (OS) in the training dataset. Only those lncRNAs with a p value of <0.005 were considered statistically significant. To construct a predictive model, each of the selected lncRNA genes was analyzed using a multivariable Cox regression model in the training dataset, with OS as the dependent variable and other clinical information as the covariables. A risk score was then computed as follows:

$$ Risk\;Score\;(RS) = \sum\limits_{i = 1}^{N} {\left( {Exp_{i} * Coe_{i} } \right)} $$

where $N$ is the number of prognostic lncRNA genes, $Exp_{i}$ is the expression value of $\ln cRNA_{i}$, and $Coe_{i}$ is the estimated regression coefficient of $\ln cRNA_{i}$ in the multivariable Cox regression analysis. This risk score model was established by taking into account the power of each of the prognostic lncRNA genes.

Using the median risk score in the training dataset as a cutoff value, NSCLC patients in each dataset were divided into high- and low-risk groups. Kaplan–Meier survival analyses were performed to test the equality for survival distributions in different groups for each NSCLC cohort, and statistical significance was assessed using the two-sided log-rank test. Additionally, a multivariable Cox regression analysis and data stratification analysis were performed to test whether the risk score was independent of other clinical features within the available data. The time-dependent receiver operating characteristic (ROC) curve was also used to compare the sensitivity and specificity of the survival prediction of the lncRNA expression-based risk score in the training dataset. Area under the curve (AUC) value was calculated from the ROC curve. All analyses were performed using R software and Bio-conductor. Significance was defined as p < 0.05.

### Bioinformatics analysis of lncRNA gene function prediction

The co-expressed relationships between the prognostic lncRNAs and protein-coding genes were computed using Pearson correlation coefficients. Gene ontology (GO) and Kyoto encyclopedia of genes and genomes (KEGG) enrichment analyses of the co-expressed protein-coding genes with prognostic lncRNAs were performed to predict the biological function of prognostic lncRNAs using the DAVID Bioinformatics Tool (version 6.7), which is a commonly used functional annotation tool that can assess over-representation of functional categories among a gene set of interest [37]. Enrichment analysis was carried out using the functional annotation chart and functional annotation clustering options, and was limited to KEGG pathways and GO terms in the “Biological Process” categories. Functional annotation with *p* value of <0.05 and an enrichment score of >2 were considered significant.

## Results

### Derivation of an eight-lncRNA prognostic signature from the training dataset

The NSCLC patient cohort from GSE37745 (n = 196), including the relatively large patient sample size and relatively overall clinical information, was selected as training dataset to explore the association between lncRNA expression and OS of NSCLC patients. We first conducted a univariate Cox proportional hazards regression analysis of the lncRNA expression data with OS as the dependent variable, and identified a set of eight lncRNAs as prognostic lncRNAs which were significantly correlated with patients’ OS (p value of <0.005). Table 2 shows a list of these eight prognostic lncRNAs along with important variable information. Of the eight lncRNAs, the higher expression level of lncRNA *RP11*-*21L23*.*2*, *GPR158*-*AS1*, *RP11*-*701P16*.*5* and *RP11*-*379F4*.*4* was associated with shorter OS (coefficient >0), and the higher expression levels of the remaining four lncRNAs (*CTD*-*2358C21*.*4*, *RP11*-*94L15*.*2*, *KCNK15*-*AS1* and *AC104134*.*2*) were associated with longer OS (coefficient < 0). Then we further examined whether these eight prognostic lncRNAs are differentially expressed between cancer and normal lung tissue. The lncRNA differential expression analysis was performed for GSE18842 dataset (including 46 tumor and 45 normal lung tissue specimens) (http://www.ncbi.nlm.nih.gov/geo/query/acc.cgi?acc=GSE18842) [38] obtained from GEO database. We found that five of eight prognostic lncRNAs showed significant expression differences between tumor and normal lung tissue (Mann–Whitney U test p < 0.05) (Additional file 1: Figure S1), demonstrating that these selected prognostic lncRNAs are associated with lung cancer.

**Table 2:** Eight lncRNAs significantly associated with the overall survival of NSCLC patients in the training set (n = 196)

Ensembl id	Gene symbol	Chromosomal position	*P* value^a^	Hazard ratio^a^	Coefficient^a^
ENSG00000261578.1	*RP11*-*21L23*.*2*	Chr11: 76,800,364-76,804,555(+)	2.57E−05	1.374	0.318
ENSG00000261731.2	*CTD*-*2358C21*.*4*	Chr16: 31,709,113-31,711,984(−)	2.22E−04	0.725	−0.322
ENSG00000264198.2	*RP11*-*94L15*.*2*	Chr17: 39,757,715-39,763,836(−)	2.88E−04	0.738	−0.303
ENSG00000233642.1	*GPR158*-*AS1*	Chr10: 25,158,072-25,176,276(−)	4.52E−04	1.334	0.288
ENSG00000244558.3	*KCNK15*-*AS1*	Chr20: 44,694,892-44,746,021(−)	1.27E−03	0.750	−0.287
ENSG00000225420.1	*AC104134*.*2*	Chr2: 88,538,720-88,575,610 (+)	1.85E−03	0.760	−0.274
ENSG00000251230.3	*RP11*-*701P16*.*5*	Chr4: 184,844,585-184,855,751(−)	2.01E−03	1.301	0.263
ENSG00000240207.4	*RP11*-*379F4*.*4*	Chr3: 158,732,263-158,784,070(+)	2.80E−03	1.433	0.360

^a^Derived from the univariable Cox regression analysis in the training set.

### An eight-lncRNA signature predicts survival of NSCLC patients in the training dataset

**Figure 1:**
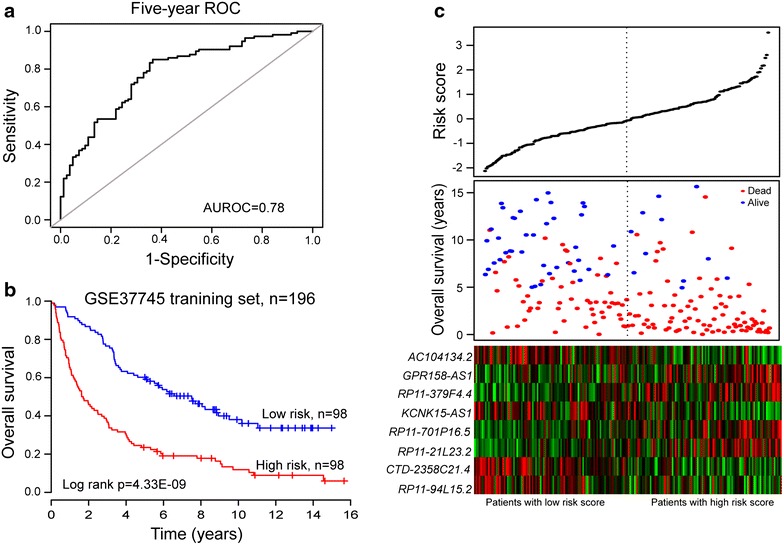
The eight-lncRNA signature-focused risk score in prognosis of overall survival in the GSE37745 patient set.** a** Receiver operating characteristic (ROC) analysis of the risk scores for overall survival prediction in the training dataset. The area under the curve (AUC) was calculated for ROC curves, and sensitivity and specificity were calculated to assess score performance.** b** The Kaplan–Meier curve for overall survival of two patient groups with high- and low-risk scores in the GSE37745 training set (n = 196). The differences between the two curves were evaluated by the two-sided log-rank test.** c** The eight lncRNA-based risk score distribution, patients’ survival status and heatmap of the eight lncRNA expression profiles. The* black dotted line* represents the cutoff value of the risk score derived from the training set which separated patients into high- and low-risk groups.

To investigate whether the eight-lncRNA signature could provide an accurate prediction of OS in NSCLC patients, the expression data of these eight lncRNAs and other clinical features were fitted into a multivariable Cox regression model as covariates of the training dataset. A risk score was generated for each patient in the training dataset according to the risk-score model (see “Methods”) as follows: Risk score = (0.306 × expression value of *RP11*-*21L23*.*2*) + (−0.314 × expression value of *CTD*-*2358C21*.*4*) + (−0.252 × expression value of *RP11*-*94L15*.*2*) + (0.288 × expression value of *GPR158*-*AS1*) + (−0.271 × expression value of *KCNK15*-*AS1*) + (−0.299 × expression value of *AC104134*.*2*) + (0.284 × expression value of *RP11*-*701P16*.*5*) + (0.321 × expression value of *RP11*-*379F4*.*4*). To evaluate how well the risk score predicts the 5-year survival, the various cutoff values were evaluated using time-dependent ROC curve (Figure 1a) which is commonly used for revealing the predictive accuracy of a model [39, 40]. In the training dataset, AUC for the eight-lncRNA signature prognostic model was 0.78 at an OS of 5 years, demonstrating the better performance for survival prediction of the lncRNA expression-based risk score in the training dataset. All patients in the training dataset were then ranked according to their risk score, and divided into either the high- or low-risk group using the median risk score as the cutoff point. According to this cutoff value, patients were divided into either a high-risk group (n = 98) or a low-risk group (n = 98). Patients in the high-risk group had a significantly shorter OS than those in the low-risk group (median OS 1.67 vs. 6.06 years, log-rank test p = 4.33E−09). Kaplan–Meier curves for the high- and low-risk groups in the training dataset (n = 196) are shown in Figure 1b. In detail, OS rates of patients in the high-risk group were 30.6% at 4 years, 19.1% at 6 years, 17.8% at 8 years and 11.9% at 10 years, versus 63.3, 53.8, 46.3 and 38% in the low-risk group, respectively.

A significant association between the eight-lncRNA signature risk score and OS was observed in the univariable Cox regression model (Table 3). The hazard ratios of the eight-lncRNA signature risk score of the high-risk group versus that of the low-risk group for OS was 2.641 [p < 0.001; 95% confidence interval (CI) 1.887–3.697; Table 3].

**Table 3:** Univariable and multivariable Cox regression analysis of the lncRNA signature and overall survival of NSCLC patients in the training and two independent cohorts

Variables		Univariable analysis			Multivariable analysis		
		HR	95% CI of HR	*P* value	HR	95% CI of HR	*P* value
GSE37745 training set, n = 196							
Eight-lncRNA risk score							
Low risk/high risk		2.641	1.887–3.697	<0.001	2.761	1.934–3.942	<0.001
Age							
≤65/>65		1.355	0.977–1.878	0.069	1.427	1.024–1.986	0.035
Gender							
Female/male		1.096	0.789–1.523	0.585	0.913	0.640–1.303	0.616
Stage							
I		1 (reference)			1 (reference)		
II		1.220	0.793–1.875	0.366	1.169	0.758–1.802	0.479
III		1.864	1.187–2.928	0.007	1.656	1.052–2.608	0.029
IV		1.313	0.415–4.152	0.643	1.566	0.488–5.022	0.451
Subtype							
Adenocarcinoma		1 (reference)			1 (reference)		
Large cell carcinoma		0.891	0.520–1.528	0.675	0.782	0.449–1.360	0.383
Squamous cell carcinoma		1.257	0.883–1.791	0.205	0.920	0.623–1.359	0.676
GSE31210 testing set, n = 226							
Eight-lncRNA risk score							
Low risk/High risk		3.067	1.471–6.395	0.003	2.643	1.263–5.528	0.010
Age							
≤65/>65		2.584	1.313–5.083	0.006	3.685	1.800–7.544	<0.001
Gender							
Female/male		1.519	0.780–2.955	0.219	1.143	0.402–3.246	0.802
Smoking status							
No/Yes		1.637	0.837–3.201	0.15	1.388	0.482–3.996	0.544
Stage							
I/II		4.232	2.175–8.236	<0.001	4.363	2.161–8.811	<0.001
GSE50081 testing set, n = 181							
Eight-lncRNA risk score							
Low risk/high risk		1.795	1.127–2.859	0.014	1.752	1.014–3.026	0.044
Age							
≤65/>65		1.559	0.932–2.608	0.090	1.316	0.752–2.303	0.336
Gender							
Female/male		1.934	1.190–3.143	0.008	1.743	1.011–3.005	0.046
Smoking status							
No/Yes		1.387	0.659–2.916	0.389	1.054	0.476–2.333	0.897
Stage							
I/II		1.689	1.049–2.718	0.031	2.359	1.379–4.034	0.002
Subtype							
Adenocarcinoma		1 (reference)			1 (reference)		
Large cell carcinoma		1.326	0.479–3.671	0.587	1.094	0.376–3.184	0.870
Squamous cell carcinoma		0.791	0.456–1.371	0.403	0.479	0.241–0.952	0.036

The distribution of risk score, survival status and prognostic lncRNA expression in 196 patients of the training dataset are shown in Figure 1c. Of these eight prognostic lncRNAs, the high expression level of lncRNA *RP11*-*21L23*.*2*, *GPR158*-*AS1*, *RP11*-*701P16*.*5* and *RP11*-*379F4*.*4* was associated with high risk, while the remaining four lncRNAs (*CTD*-*2358C21*.*4*, *RP11*-*94L15*.*2*, *KCNK15*-*AS1* and *AC104134*.*2*) were shown to be protective. NSCLC patients with high prognostic scores tended to express high-risk lncRNAs, whereas those with low prognostic scores tended to express protective lncRNAs. Moreover, more deaths were noted for NSCLC patients with high-risk scores than for those with low-risk scores.

### Validation of the eight-lncRNA signature for survival prediction in the testing GSE31210 dataset

**Figure 2:**
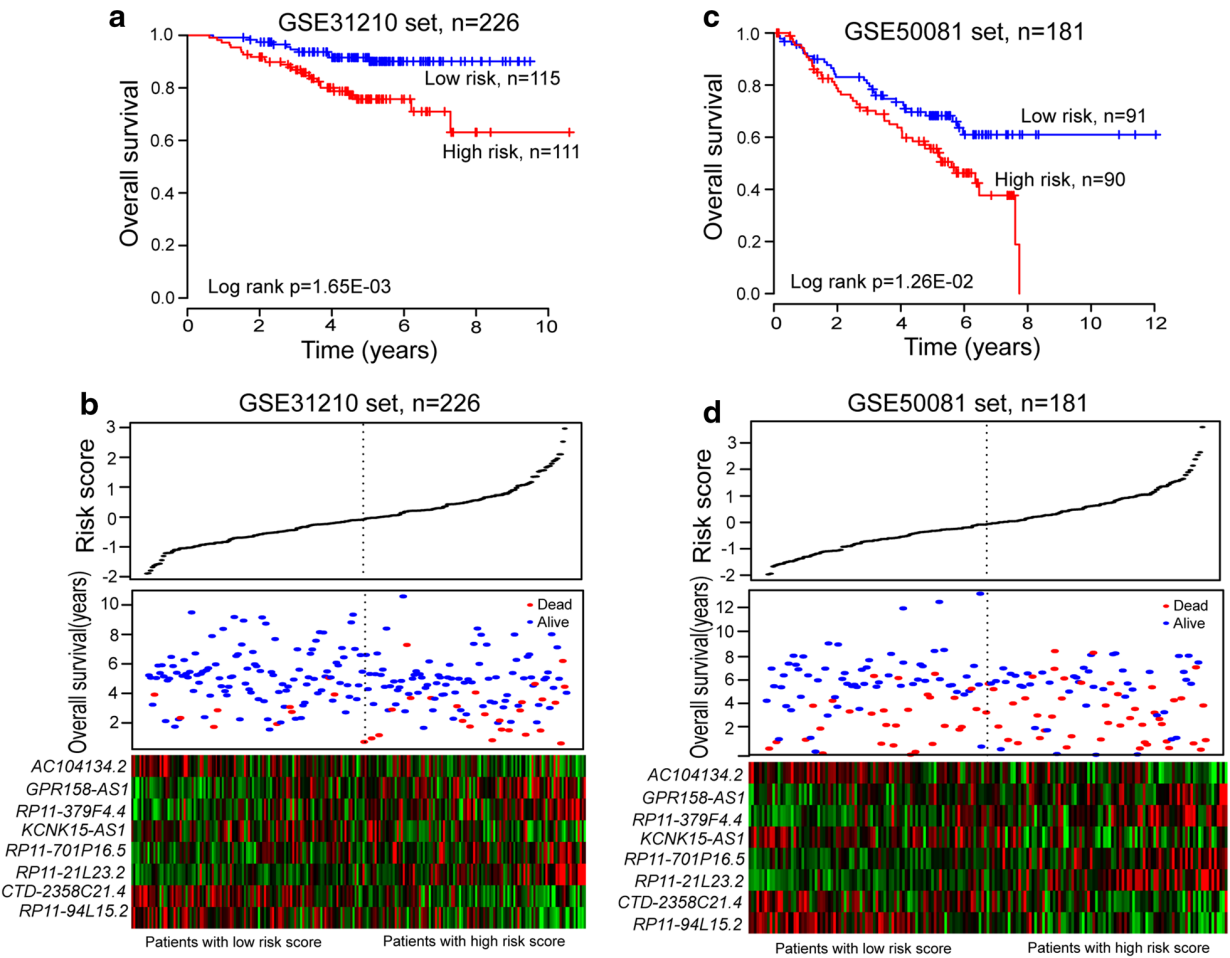
The eight-lncRNA signature-focused risk score in prognosis of overall survival in additional validation datasets.** a** Kaplan–Meier survival curves were plotted for GSE31210 (n = 226).** b** The eight lncRNA-based risk score distribution, patients’ survival status and heatmap of the eight lncRNA expression profiles were analyzed in the GSE31210.** c** Kaplan–Meier survival curves were plotted for GSE50081 (n = 181).** d** The eight lncRNA-based risk score distribution, patients’ survival status and heatmap of the eight lncRNA expression profiles were analyzed in the GSE50081.

To validate the prognostic power of the eight-lncRNA signature for survival prediction, the constructed expression-defined lncRNA prognostic model was also evaluated in the testing GSE31210 dataset. The same prognostic risk score model obtained from the training dataset was used to calculate the eight-lncRNA signature-based risk scores for 226 patients in the entire GSE31210 dataset. The cutoff value of the risk score derived from the training dataset without re-estimating parameters was used for the testing dataset to classify the patients into either a high-risk group (n = 111) or a low-risk group (n = 115). Patients with high-risk scores exhibited poorer OS than those with low-risk scores (median OS 4.45 vs. 5.08 years, log-rank test p = 1.65E−03). Kaplan–Meier curves for the high- and low-risk groups in the testing dataset are shown in Figure 2a. The OS rate of patients in the high-risk group was 91.7% at 2 years and 78.7% at 4 years, versus 97.4 and 91.5% in the low-risk group, respectively. A significant association between the eight-lncRNA signature risk score and OS in the univariable Cox regression model was observed. The hazard ratios of the eight-lncRNA signature risk scores of the high-risk group versus the low-risk group for OS was 3.067 (p = 0.003; 95% CI 1.471–6.395; Table 3).

The distribution of patient lncRNA risk score, survival status and prognostic lncRNA expression in 226 patients of the GSE31210 dataset are shown in Figure 2b, revealing the similar results observed in the GSE37745 training dataset.

### Further validation of the eight-lncRNA signature in another independent dataset

To investigate the reproducibility of the eight-lncRNA signature in predicting OS of NSCLC patients, the prognostic power of the eight-lncRNA signature for prediction of survival was further validated in another independent NSCLC cohort of 181 patients whose expression and survival data were obtained from GEO GSE50081. The clinical feature of this independent NSCLC cohort is shown in Table 1. Patients in this independent NSCLC cohort were classified into either a high-risk group (n = 90) or a low-risk group (n = 91) according to the cutoff value of risk scores obtained from the training dataset. The median OS of the high-risk group for the GSE50081 dataset is 4.29 years, whereas that of the low-risk group is 4.99 years (log-rank test p = 1.26E−02). Kaplan–Meier curves for the high- and low-risk groups within the independent GSE50081 cohort is shown in Figure 2c. Further univariable Cox regression analysis revealed that the high-risk scores of eight-lncRNA signature was significantly associated with lower OS of patients in GSE50081 dataset (p = 1.40E−02; HR = 1.795, 95% CI 1.127–2.859; Table 3). Figure 2d shows the distribution of patient risk scores, the survival status and prognostic lncRNA expression in the independent GSE50081 NSCLC cohort, ranked according to the prognostic risk score values for the eight-lncRNA signature, which were similar to those observed in the training and GSE31210 datasets.

### Survival prediction by the eight-lncRNA signature is independent of clinical features

**Figure 3:**
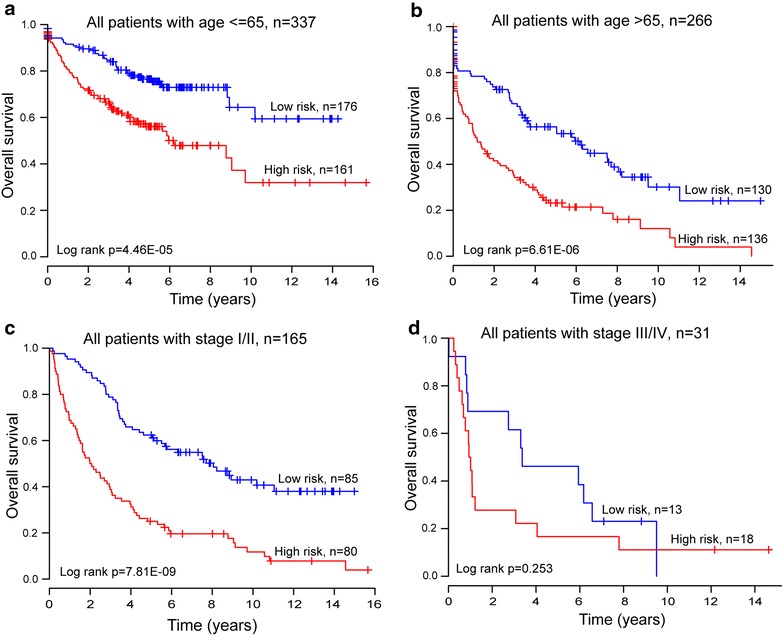
Survival analyses of all patients with available age or tumor stage information using the eight-lncRNA signature.** a** Kaplan–Meier survival curves for younger patients with NSCLC (age ≤65, n = 337).** b** Kaplan–Meier survival curves for elder patients with NSCLC (age >65, n = 226).** c** Survival prediction in early stage patients: Kaplan–Meier survival curves for all patients with stage I and II (n = 165).** d** Survival prediction in late stage patients: Kaplan–Meier survival curves for all patients with stage III and IV (n = 31).

To assess whether the prognostic power of the eight-lncRNA signature for prediction of survival was independent of other clinical features, multivariable Cox regression analysis was performed using the lncRNA signature-based risk score and other clinical features, including age, gender, smoking status, tumor stage and subtype, which were used as covariates. The results of multivariable Cox regression analysis from three NSCLC patients datasets showed that the prognostic power of the eight-lncRNA signature risk score (high-risk group vs. low-risk group, HR = 2.761, 95% CI 1.934–3.942, p < 0.001 for GSE37745; HR = 2.643, 95% CI 1.263–5.528, p = 0.01 for GSE31210; HR = 1.752, 95% CI 1.014–3.026, p = 0.044 for GSE50081) for prediction of survival was indeed independent of these clinical features (Table 3). We also found that the two clinical factors, age and stage, also affected overall survival of patients. So, a data stratification analysis was performed according to age and stage. The three GEO datasets (GSE37745, GSE31210 and GSE50081), which included a total of 603 patients, were stratified by age into either a younger stratum (age ≤65) or an elder stratum (age >65). The results of stratified analysis showed effective prognostic power in both the younger and elder patient groups. The eight-lncRNA signature could classify patients within each age stratum into either high- or low-risk groups with significantly different OS (log-rank test p = 4.46E−05 for the younger patient group and p = 6.61E−06 for the elder patient group) (Figure 3a, b), which suggested that the prognostic power of the eight-lncRNA signature was also age-independent. Then the patients of early (stage I and II) and late (III and IV) stage for GSE37745 dataset were grouped into two separate groups. The stratified analysis was further performed in early and late stage patients to evaluate whether the eight-lncRNA signature could predict survival of patients for different clinical stage. The log-rank test of early stage patients showed that within stage I and II, the eight-lncRNA signature could further subdivide them into either a high-risk group with shorter survival or a low-risk group with longer survival (median OS 2.03 vs. 8.05 years, log-rank test p = 7.81E−09) (Figure 3c). Difference for OS between high-risk group (n = 18) and low-risk group (n = 13) also was observed for late stage patients (median OS 0.975 vs. 3.367 years) (Figure 3d), although the log-rank p value is 0.253 which was above the 0.05 significance level.

### Functional characterization of the eight prognostic lncRNAs

**Figure 4:**
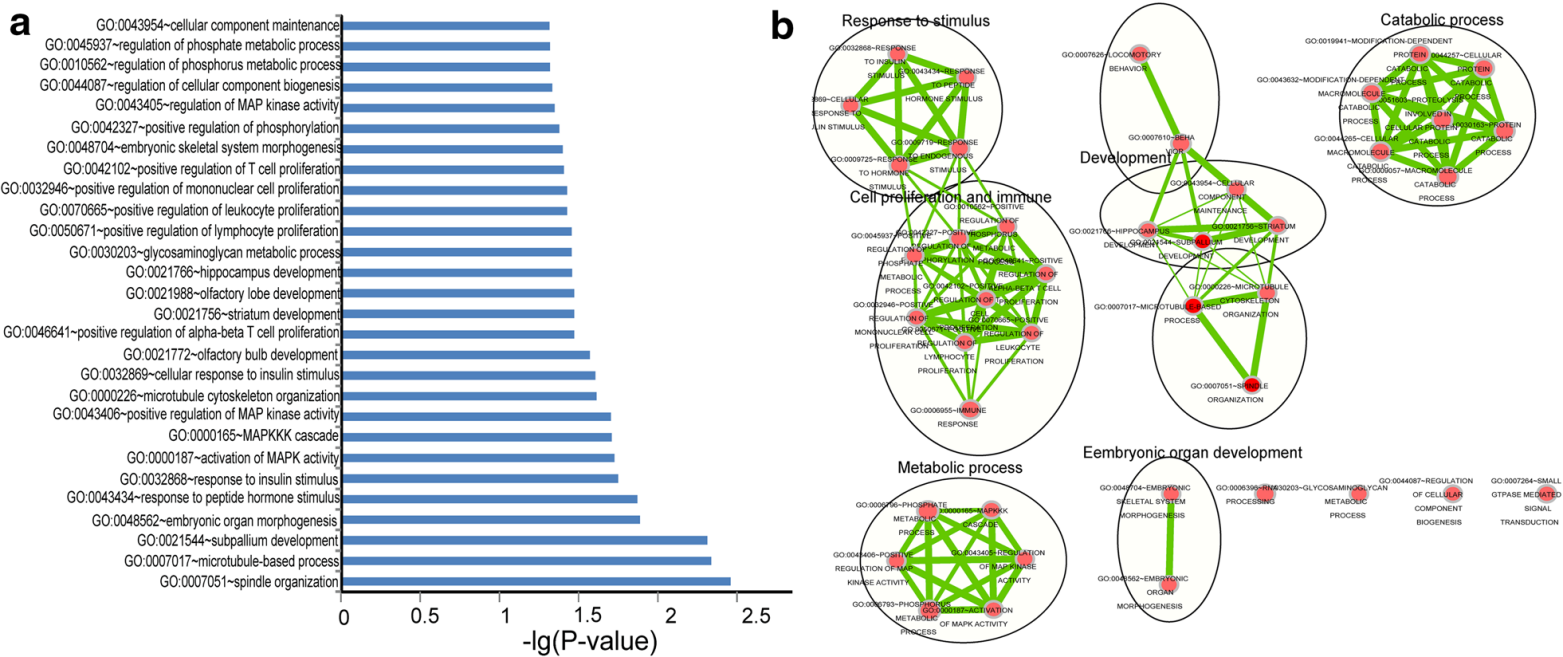
Functional enrichment results of the co-expressed protein-coding genes with prognostic lncRNAs.** a** Significantly enriched GO terms of the co-expressed protein-coding genes with prognostic lncRNAs. **b** The functional enrichment map of GO terms.* Each node* represents a GO term, which are grouped and annotated by GO similarity. A* link* represents the overlap of shared genes between connecting GO terms.* Node size* represents the number of gene in the GO terms.* Color intensity* is proportional to enrichment significance.

To further investigate the potential biological roles involving the eight prognostic lncRNAs, the co-expressed relationships between the expression of eight lncRNAs and those of the protein-coding genes were computed using Pearson correlation coefficients in the GSE37745 dataset of 196 patients. The expression of 679 protein-coding genes were highly correlated with that of at least one of the eight signature lncRNAs (Pearson correlation coefficient >0.40). GO and KEGG pathway function enrichment analysis for these co-expressed protein-coding genes was then performed, using the whole human genome as the background. The results showed that four genes (*GATA6*, *CRISPLD2*, *CFTR2* and *CLPTM1L*) have been proven to be involved in lung cancer. GO functional annotation suggested that 679 protein-coding genes were significantly enriched in 28 GO terms (Figure 4a). These significant GO terms were organized into an interaction network with similar functions using the Enrichment Map [41] plugin in Cytoscape [42]. Several clusters of functionally related GO terms were observed including organ development and cell proliferation and immune, response to stimulus, catabolic and metabolic process (Figure 4b). Taken together, these results implied that the eight lncRNAs might be involved in tumorigenesis through interacting with protein-coding genes that affect the tissue/organ development and other important biological processes.

## Discussion

During the past few decades, considerable efforts have been made toward the development of gene-expression-based diagnostic and prognostic biomarkers for lung cancer at the protein-coding genes and miRNAs levels [43, 44]. However, accumulating evidence suggested that lncRNA are involved in oncogenic and tumor suppressive pathways have opened the door for this new biomarker. Transcriptional profiling analyses have discovered a number of tissue-specific lncRNAs in normal tissues and dysregulated lncRNAs in a variety of human cancers [11, 45], and highly aberrant expression of dysregulated lncRNAs is associated with tumorigenesis [17]. Furthermore, these dysregulated lncRNAs have already shown great potential as novel molecular biomarkers for diagnosis, prognosis and treatment of cancer. More recently, several studies conducted array-based transcriptional analyses of lncRNAs and functionally characterized cancer subtype-associated lncRNAs in breast cancer and lung cancer, proposing a novel clinical implication for lncRNAs as valuable biomarkers for prediction of response to treatment as well as patient outcome [27, 46]. Compared to protein-coding genes, the advantage of lncRNAs as molecular biomarkers is that lncRNA expression is more closely associated with its biological function and tumor status [16, 47]. However, to date, expression profile-based prognostic lncRNA signature for prediction of survival of NSCLC patients has not been investigated.

Recently, several studies have reported that lncRNA expression profiles can be obtained from publicly available, custom-designed DNA microarrays by re-annotating the array probes [19, 25, 26, 35, 47]. In this study, microarray probe re-annotation was used to repurpose the publicly available human Affymetrix microarray data (HG-U133 Plus 2.0) and subsequently obtain lncRNA expression profiles of 603 NSCLC patients from GEO. To identify lncRNAs with prognostic value in NSCLC, survival analysis was performed by integrating lncRNA expression profiles and clinical information in a large cohort of NSCLC patients. An expression pattern of eight lncRNAs was found to be significantly associated with OS of NSCLC patients in the GSE37745 training dataset. Further ROC analysis demonstrated good performance for predicting 5-year OS. A prognostic risk score model was developed based on the expression data of these eight lncRNAs and weighted by the estimated regression coefficients from multivariable Cox regression analysis. With this eight-lncRNA signature, patients in the training dataset with high-risk scores tended to have lower OS than those with low-risk scores. The separation between survival curves for high- and low-risk patients of the training dataset used for model development was observed. A previous simulation study revealed that a prognostic model can also be developed that is significantly associated with survival time in the training dataset when using completely random gene expression profiles [48]. To evaluate the robustness and reproducibility of the prognostic power of the eight-lncRNA signature, it was also tested in the non-overlapping two other independent NSCLC patient cohorts (GSE31210 and GSE50081) using the same model and criteria as those from the training dataset. In these tests, the prognostic power was also strong, indicating that the eight-lncRNA signature demonstrated good reproducibility and robustness for the NSCLC patients.

Several studies have observed different clinical characteristics and survival time among different age groups of NSCLC patients [49–51]. Multivariable Cox regression analysis was thus used to assess the independence of the eight-lncRNA signature in predicting OS. With age, gender, smoking status, stage and subtype as covariables in the regression analysis, risk score of the eight-lncRNA signature was found to have maintained an independent correlation with OS. In the stratified analysis, the eight-lncRNA signature showed prognostic power for different age groups, in which patients belonging to the same age group could be classified into high- and low-risk groups with significantly different survival prospects, indicating that the prognostic value of the eight-lncRNA signature was independent of age of the NSCLC patients. In lung cancer, clinicopathological parameters like tumor histology, staging and localization of metastases determine patients’ outcome [52]. Since tumor stage and subtype data was only available for the GSE37745 patient dataset, multivariate Cox regression analysis and stratified analysis were performed to assess the stage- and subtype-independence of prognostic power of the eight-lncRNA signature. The eight-lncRNA signature was indeed found to be stage-dependent in NSCLC patients, and its prognostic power was significant in stage I and II patients, in which all patients in stage I and II could be separated into high- and low-risk groups with significantly different survival. However, the eight-lncRNA signature achieved a p value of 0.253 for OS prediction of late stage patients, which was above the 0.05 significance level, suggesting that patients with early stage cancer may benefit significantly from this eight-lncRNA prognostic signature. Further multivariate Cox regression analysis testing tumor subtype-independence suggested that prognostic power of the eight-lncRNA signature is independent of tumor subtype. Taken together, these results suggest that the prognostic power of the eight-lncRNA signature for predicting OS of NSCLC patients is independent of other clinical features except for stage.

Tens of thousands of lncRNAs have been identified and predicted by large-scale transcriptome analysis in humans [53]. However, the functions of only a few lncRNAs have been well characterized, so no thorough functional annotation data is available for the eight prognostic lncRNAs in the current literature. Recent bioinformatics studies have suggested that the function of lncRNAs could effectively be predicted with the inclusion of different kinds of biological data. To increase our understanding of the biological roles of the eight prognostic lncRNAs in NSCLC, functional enrichment analysis was performed for 679 protein-coding genes co-expressed with the eight prognostic lncRNAs at the GO and KEGG pathway level. The biological processes most highly associated with the genes were organ development, cell proliferation and immune, response to stimulus, catabolic and metabolic process. In particular, several co-expressed protein-coding genes with eight prognostic RNAs were proven to participate in the NSCLC pathway. These results implied important functional roles of the eight prognostic lncRNAs in tumorigenesis.

Due to the restriction of available data, gene expression profiles of only 2,313 of the tens-of-thousands of known and predicted lncRNAs were obtained. However, the prognostic power of the eight-lncRNA signature uncovered in this study for predicting OS consistently observed in multiple independent datasets. Moreover, the incompleteness and low coverage of available lncRNA-related datasets are common when by studying lncRNA-disease associations. Although the functions of these eight lncRNAs have been inferred by bioinformatics analysis, the biological roles of these eight lncRNAs in tumorigenesis are still not clear and should be investigated in further experimental studies. With the rapid increase of lncRNA-related studies, more comprehensive lncRNA will become available, and lncRNA biomarker development for clinical prognostic evaluation of NSCLC should increase.

## Conclusions

In summary, by examining lncRNA expression profiles of patients with NSCLC, our study identified eight lncRNAs associated with overall survival of NSCLC patients. A prognostic lncRNA signature was developed based on the expression patterns of these eight lncRNAs in the training dataset to predict the overall survival, and subsequently was validated in other two independent datasets. Further analysis demonstrated that the prognostic power of the eight-lncRNA signature for prediction of survival was independent of other clinical features. Our results suggested that the eight-lncRNA signature may be an effective independent prognostic molecular biomarker in the prediction of NSCLC patient survival.

## Additional file

Additional file 1:
**Figure S1.** The boxplot of expression level of eight prognostic lncRNAs in lung cancer and control samples.

## Abbreviations

AUC: area under the ROC curve
CeRNA: competing endogenous RNA
CI: confidence interval
CRC: colorectal cancer
GEO: gene expression omnibus
GO: gene ontology
HR: hazard ratio
KEGG: Kyoto encyclopedia of genes and genomes
LncRNAs: long non-coding RNAs
MALAT1: metastasis-associated lung adenocarcinoma transcript 1
NPC: nasopharyngeal carcinoma
NSCLC: non-small cell lung cancer
OS: overall survival
OSCC: oesophageal squamous cell carcinoma
RMA: robust multichip average
ROC: receiver operating characteristic
SD: standard deviation
