# Supplementary figures and images for: A potential signature of eight long non-coding RNAs predicts survival in patients with non-small cell lung cancer
Source: J Transl Med. 2015 Jul 17;13:231. doi: 10.1186/s12967-015-0556-3 (PMC4504221; doi:10.1186/s12967-015-0556-3)

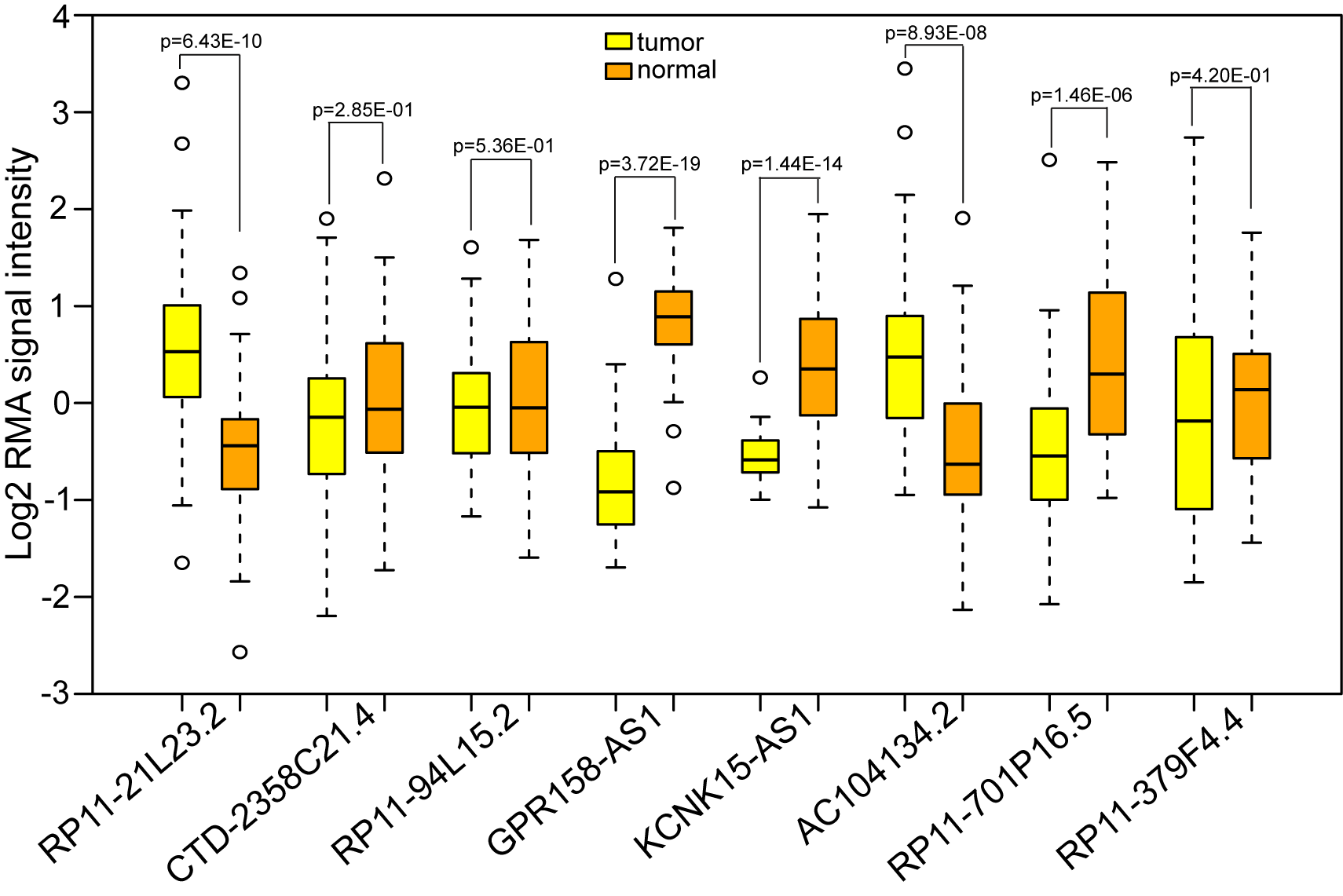

Supplement: Additional file 1: — Figure S1. The boxplot of expression level of eight prognostic lncRNAs in lung cancer and control samples. [file 12967_2015_556_MOESM1_ESM.tiff]
